# Functional Nanomaterials for Optoelectronics and Photocatalysis

**DOI:** 10.3390/nano13192694

**Published:** 2023-10-03

**Authors:** Protima Rauwel, Erwan Rauwel

**Affiliations:** 1 Institute of Forestry and Engineering Sciences, Estonian University of Life Sciences, 51006 Tartu, Estonia; erwan.rauwel@emu.ee; 2 Institute of Veterinary Medicine and Animal Sciences, Estonian University of Life Sciences, 51006 Tartu, Estonia

The present energy crisis has encouraged the use of energy-efficient devices and green energy sources. In addition to their energy-efficient operation, it is now essential that the production of these devices is cost-effective. Devices requiring energy-efficient operation and production include light emitting diodes (LEDs) applied to general lighting systems or for specific applications in electronic devices. With regard to the production of energy, cost-effective and new materials are being intensively investigated. The new generation of devices consists of hybrid materials and nanomaterials, involving polymers coupled with inorganic counterparts. The advantages of these hybrid materials include lower production costs, an overall weight reduction in the device and easier recyclability. In this regard, functional nanomaterials appear to be the most suitable choice of materials for these applications. In electronic devices, they allow miniaturization, while in energy-harvesting applications, i.e., photovoltaics and photocatalysis, they allow for more efficient energy conversion owing to the higher surface-to-volume ratio. Since the active sites for energy conversion in these nanomaterials are localized on the surface, the volume of the device is therefore reduced. Hence, the energy produced per unit mass is higher, as a lower amount of material is required in the device. Along with cost-effective production techniques, the overall device costs are therefore lowered.

The present Special Issue focuses on functional nanomaterials applied to optoelectronics and photocatalysis with several common nanomaterials to both fields, in particular ZnO. The compilation is clearly divided into three categories: (i) optoelectronics, (ii) photovoltaics and (iii) photocatalysis. In the optoelectronics section, the publication of Kabongo et al. describes the synthesis of ZnO doped with Ho, exhibiting ferromagnetic properties under microwave excitation [1]. The second publication, by Rauwel et al., reports on the combination of ZnO nanoparticles with CNT and Ag nanoparticles, and emphasizes the plasmonic effect of Ag nanoparticles in the enhancement of UV emission owing to the Burstein–Moss effect [2]. The plasmonic effect of metal nanoparticles has also been theoretically studied by Shivangi et al. in the enhancement of an SPR-based sensor device of BlueP/WS_2_-covered Al_2_O_3_-nickel nanofilms [3]. Another article, by Nagpal et al., describes the enhancement and suppression of the visible light emission of ZnO nanostructures with the addition of carbon nanotubes (CNTs) and poly(3,4-ethylenedioxythiophene) polystyrene sulfonate (PEDOT:PSS), respectively [4]. Polymers, such as Poly(methyl methacrylate) (PMMA), are also used as electron-blocking layers in a II–VIsemiconductor QLED, as described by Zvaigzne et al. [5]. Similar II-VI semiconductor materials were also grown by Hou et al. via a phosphine-free method, and their photovoltaic properties were evaluated, which marks the second topic of this Special Issue [6]. The third topic, i.e., photocatalysis, is composed of four publications. This topic can be divided into two subgroups: (i) H_2_ production and (ii) dye degradation. For H_2_ production, Xia et al. report on a heterostructure of an organic/inorganic interface of g-C_3_N_4_/LDH that can be activated under visible light radiation [7]. This Special Issue contains three publications on the study of dye degradation using nanomaterials: The first publication is a review article by Paredes et al. that surveys the Cu_3_N nanomaterials used to date [8]. The second publication by Paredes et al. describes the one-step synthesis of nanoparticle mixtures of Cu-Cu_3_N-Cu_2_O and their potential in the sunlight-driven photocatalytic degradation of azo dyes [9]. The last publication, by Hendrix et al., reports on the degradation of azo dyes using ZnO nanomaterials, and investigates, for the first time, the influence of their morphology and defect states under both UV and sunlight [10].

We wish you a pleasant read and hope that this Special Issue on “Functional Nanomaterials for Optoelectronics and Photocatalysis” will serve as a valuable resource for researchers and PhD students in the field.
